# Drug-induced stuttering: Focus on medication with Antipsychotics

**DOI:** 10.1192/j.eurpsy.2025.2091

**Published:** 2025-08-26

**Authors:** J. Marta, Â. Ferreira, A. F. Reis, T. Cardoso, C. Batista, J. Miranda, V. Vila Nova

**Affiliations:** 1 Setúbal Hospital Center, Setúbal, Portugal

## Abstract

**Introduction:**

Stuttering is a disruption in speech fluency, characterised by repetition of sounds, syllables, or words, consonant prolongations, and blockages. It can be classified as developmental or acquired, the latter being psychogenic or neurogenic. Neurogenic stuttering is often associated with brain injuries, stroke, neurodegenerative conditions, or side effects of certain medications. Drug-induced stuttering (DIS) is a recognised but rare side effect of psychotropic medications, particularly antipsychotics. Although uncommon, DIS significantly impacts quality of life, especially in males aged 20 to 40. Clinicians may mistakenly attribute the onset of stuttering to the progression of psychiatric or neurological conditions, overlooking the potential role of medication. The exact mechanism of DIS remains unclear, but it is likely related to disruptions in neurotransmitter systems.

**Objectives:**

This review aims to explore the pathophysiology and neurochemical pathways involved in antipsychotic-induced stuttering (AIS) through a literature review.

**Methods:**

A non-systematic literature review was conducted using PubMed with the keywords “psychopharmacology”, “stuttering”, and “fluency disorder”.

**Results:**

Clozapine emerges as the primary drug implicated in DIS, though cases involving olanzapine, risperidone and aripiprazole have also been reported. The pathogenesis of AIS likely involves neurotransmitter system disruptions, particularly dopamine, glutamate and serotonin, within circuits such as the cortico-basal ganglia-thalamocortical loop and white matter fiber tracts. Dopamine dysregulation appears to play a central role, as antipsychotics that block dopamine receptors may impair motor control in speech-related pathways, and additionally in the prefrontal cortex and nigrostriatal pathway. This disruption alters the balance between different brain regions, leading to deficits in motor control over the muscles involved in verbal articulation, which subsequently manifest as stuttering. Furthermore, cognitive and sensory disorders may also contribute to DIS pathogenesis.

Interestingly, stuttering improvement has been noted in some individuals with the same medications that induce it in others, reflecting the variability of dopamine’s role in different brain processes.

**Conclusions:**

DIS, particularly from antipsychotic medications, is a rare but significant clinical issue that may be overlooked. It can cause substantial social impairment, especially in psychiatric patients, who are vulnerable to stigma. Careful monitoring of medication side effects, particularly in those with no prior stuttering history, is essential to provide the best possible care. Drug withdrawal or dose adjustment is often an effective intervention for managing DIS.

**Disclosure of Interest:**

None Declared

